# The prevalence of urolithiasis in subjects undergoing computer tomography in selected referral diagnostic centers in Mogadishu, Somalia

**DOI:** 10.3389/fpubh.2023.1203640

**Published:** 2023-10-27

**Authors:** Najib Isse Dirie, Mohamed Hussein Adam, Bashiru Garba, Hassan Abdullahi Dahie, Maryan Abdullahi Sh. Nur, Fartun Yasin Mohamed, Abdirahman Khalif Mohamud, Jihaan Hassan

**Affiliations:** ^1^Department of Urology, Dr. Sumait Hospital, Faculty of Medicine and Health Sciences, SIMAD University, Mogadishu, Somalia; ^2^Department of Public Health, Faculty of Medicine and Health Sciences, SIMAD University, Mogadishu, Somalia; ^3^Faculty of Medicine and Health Sciences, SIMAD University, Mogadishu, Somalia; ^4^Department of Veterinary Public Health and Preventive Medicine, Faculty of Veterinary Medicine, Usmanu Danfodiyo University, Sokoto, Nigeria; ^5^Department of Nursing and Midwifery, Faculty of Medicine and Health Sciences, SIMAD University, Mogadishu, Somalia; ^6^Department of Obstetrics and Gynecology, Dr. Sumait Hospital, Faculty of Medicine and Health Sciences, SIMAD University, Mogadishu, Somalia; ^7^Department of Microbiology and Medical Laboratory Sciences, Faculty of Medicine and Health Sciences, SIMAD University, Mogadishu, Somalia

**Keywords:** urolithiasis, prevalence, non-contrast computed tomography, Mogadishu, Somalia

## Abstract

**Introduction and objectives:**

Somalia was predicted to be in the global stone belt with high urolithiasis prevalence. We aimed to determine the prevalence of urolithiasis and their demographic and computer tomography (CT) characteristics among subjects under CT scans in Mogadishu, Somalia. Materials and Methods: From March 2014 to November 2022, a total of 7,276 patients who underwent an abdominopelvic non-contrast CT scan for various indications were retrospectively reviewed. The mean age was 45.6 years with a standard deviation of 21.1 (range, 0.2–110 years). Patients were subdivided into two categories: adults (≥18 years) and pediatric (≤17 years).

**Results:**

Of the 7,276 patients, 1,075 (14.8%) were diagnosed with urolithiasis. Among those with urolithiasis, 702 (65.3%) were male patients, and 373 (34.7%) were female patients. Among them, adults accounted for 92.7%, while children were 7.3%. Renal stones (nephrolithiasis) were the most common, representing 57% followed by ureteric stones at 35.5%, making upper urinary stones 92.5%. Approximately 70 patients (6.5%) had bladder stones; of these, 26 of them (37%) were accompanied by benign prostatic hyperplasia (BPH). There were 10 urethral stones (0.9%) recorded in the study, all were found in male patients, 8 localized in prostatic urethra, and 2 in the bulbar urethra. The overall mean stone size was 13.2 mm, and 60% of them ranged from 5 to 22 mm. Only 24% of the patients were asymptomatic. Single stones were almost 70%, while staghorn calculi were 8.2%. More than 60% of the patients with urolithiasis showed some degree of hydronephrosis ranging between mild to severe.

**Conclusion:**

A CT scan-based urolithiasis prevalence indicates 14.8% in Mogadishu, Somalia, and these results are consistent with the probability calculation of the weights-of-evidence (WofE) methodology based on several risk factors including temperature, climate change, mineral deposit, drinking water quality, and distribution of carbonated rocks. Considering the high prevalence of the disease, Somalia needs to invest more in prevention and treatment facilities while also training urologists that are capable of utilizing minimally invasive techniques in the country.

## Introduction

Although some urinary stones can be clinically silent for many years, urolithiasis generally is a painful and severe disease that can cause serious renal damage and ultimately lead to renal failure (1). The lifetime recurrence of urolithiasis was reported to be very high (1). However, the prevalence varies among different regions around the world. According to the existing literature, the variations in urolithiasis prevalence depend on many factors such as age, sex, diet, genetics, water intake, climate, and geographic location (2). Regions with high annual temperatures tend to have higher prevalence and incidence of urinary stone disease (3). Somalia is an arid or semi-arid country located in the east most corner of Africa generally known as the Horn of Africa. The country is characterized by a tropical, hot, and most of the time dry climate where the annual mean temperature averages is approximately 30°C (4).

The urolithiasis study trends demonstrated increased overall prevalence globally. For instance, in the United States (US), Chewcharat et al. (5) reported an increase of kidney stone prevalence up to 10.1% between the 2007 and 2016 period, compared to 5.2 and 3.8% prevalence earlier reported in periods between 1988–1994 and 1976–1980, respectively (6). In South America, Argentina reported an overall 4% of urolithiasis prevalence in the city of Buenos Aires (7). In Asia, the prevalence varies among different countries, for example, mainland China, Taiwan, Japan, Iran, and South Korea (Seoul) recorded 4, 9.6, 10.8, 5.7, and 3.5%, respectively (2). On the other hand, it is widely reported that men are more likely to develop nephrolithiasis than women, while the prevalence of the urinary stone disease increases with age peaking in the ages between 60 and 69 years old (8).

In contrast, there is a huge data limitation from Sub-Saharan Africa regarding urolithiasis prevalence and treatment; therefore, the prevalence of the disease is not exactly known. Only a few hospital-based small-scale studies have reported incidences of the disease (1, 9, 10). For instance, in Ethiopia, Mohammed et al. reported 30% urolithiasis diagnosis from 824 urologic admissions between July 2016 and December 2017 at a university teaching hospital in Addis Ababa (10).

Somalia is a country emerging from a long-term civil war and the recent impact of the COVID-19 global pandemic (11). The country is currently dealing with a severe drought across the Greater Horn of Africa region leading to a severe humanitarian and health crisis (12, 13). Furthermore, the lack of strong health systems created a huge disease information gap in the country especially non-communicable diseases which are highly under-reported because the country is having to deal with the humanitarian health crisis including maternal health, mental health, and infectious diseases (13). To the best of our knowledge, there are no currently available published studies addressing the prevalence and the overall status of urinary stone disease in Somalia. Hence, the aim of this study was to determine the prevalence of urinary tract stones and their characteristics among inpatients undergoing CT scans in Mogadishu, Somalia.

## Materials and methods

### Study design and setting

This was a 9-year (March 2014 to November 2022) retrospective hospital-based study of all patients who underwent abdominopelvic, pelvic, and/or abdominal non-contrast computer tomography (NCCT) scans at three major diagnostic centers to determine the prevalence of urinary stone disease regardless of their age and symptoms. The study was undertaken after obtaining ethics approval and permission from the Institutional Review Board of SIMAD University, Mogadishu, Somalia (Ref: 2023/SU-IRB/FMHS/P006). Electronic databases of Kamil diagnostic center (The scan used; Neosoft 64 slice, Shenyang, China), Sahan diagnostic center (The scan used; Siemens 128 slice, Erlangen, Germany), and Dr. Sumait hospital (The scan used; 160 slice Canon, Ōtawara, Tochigi, Japan), Mogadishu, Somalia, were obtained to identify the patients of which their scans reported with urinary stone disease. CT scans done with oral or/and IV contrast were excluded from the study. The participating institutions Kamil and Sahan diagnostic centers are the two major referral imaging diagnostic centers in Mogadishu, Somalia, while Dr. Sumait hospital is the university teaching hospital affiliated with SIMAD University, Mogadishu, Somalia.

### Patient search strategy and characterization

To identify urolithiasis patients, key radiology search terms used by each of the centers were inputted into the databases, and search results were retrieved. Furthermore, the retrieved results of all the patents based on the search strategy were thoroughly reviewed for the confirmation of the diagnosis of urolithiasis.

Cases were selected from individuals who had confirmed diagnosis of urolithiasis after undergoing abdominopelvic scans. The indications for the scan ranged from abdominal, colicky, and flank pain, with a fraction being from routine medical check-ups (asymptomatic cases). Individual patients’ clinical information associated with urolithiasis, such as indication for the scan, age (adults: ≥18 years and children: ≤17 years), gender, organ involved, location of the stone, laterality, stone size (mm), number of stones, percentage of staghorn calculi, hydronephrosis and its severity, urologic concomitant diseases, and other co-existing diagnosis, was all collected. In addition, radiology reports on clinical history and type of imaging done were reviewed and used as guide for the confirmation or exclusion of urolithiasis. Incidences of urolithiasis were classified based on the affected organ, location, and number of stones and sites (whether bilateral or otherwise).

### Data analysis

The raw data were checked for consistency and completeness prior to statistical analyses using IBM, SPSS V26. Data with categorical variables were summarized into frequencies and percentages, while those with continuous variables were expressed as arithmetic mean ± standard deviation (SD).

## Results

Among 7,276 patients who underwent abdomen, pelvic, or kidney, ureter, bladder (KUB) NCCT scans during the study period, 1,075 (14.8%) were diagnosed with urinary tract stones (Table 1). The total number of male patients in the whole study was 3,755 (51.6%), of which 702 were diagnosed with urolithiasis making the prevalence within the male gender 18.7%. In contrast, the total female patients accounted for 3,521 (48.4%), of which 373 were diagnosed with urolithiasis making the prevalence 10.6% within the female gender. The majority of the study population were adults (>18 years of age) accounting for 91.5%, while the children (≤17 years of age) were 8.5% with 14.9 and 12.8% of urolithiasis cases, respectively. Concerning the centers where the data were obtained, the Kamil diagnostic center accounted for up to 95.3% (see Table 1).

**Table 1:** Overall study distribution and urolithiasis prevalence.

Parameters		Overall study patients (%)	No. of urolithiasis patients (%)
Urolithiasis prevalence		7,276 (100)	1,075 (14.8)
Diagnostic centers	Kamil diagnostic	6,937 (95.3)	1,006 (14.5)
	Sahan diagnostic	252 (3.5)	45 (17.8)
	Dr. Sumait hospital	87 (1.2)	24 (27.6)
Gender	Male	3,755 (51.6)	702 (18.7)
	Female	3,521 (48.4)	373 (10.6)
Age groups	Adult (≥18 years)	6,660 (91.5)	996 (14.9)
	Pediatric (<18 years)	616 (8.5)	79 (12.8)

The overall 1,075 subjects with urolithiasis contained 702 (65.3%) male patients and 373 (34.7%) female patients with a mean age of 45.6 years (range, 0.2–110 years) and 21.1 standard deviation (SD). Children (≤17 years) accounted for 7.3% of those with urolithiasis, while adults (>18 years) were 92.7%. Renal stones (nephrolithiasis) were the most common problem, representing 57% followed by ureteric stones accounting for 35.5%, making upper urinary stones 92.5%. Approximately 70 patients (6.5%) had bladder stones; of these, 26 of them (37%) were accompanied by benign prostatic hyperplasia (BPH). In other words, there were 49 patients with BPH, and of these, 26 of them (53%) had bladder stones. There were 10 urethral stones (0.9%) recorded in the study, where all were found in male patients, 8 localized in prostatic and 2 in bulbar urethra. With regard to the stone size, 60% of the urinary stones were 5–22 mm, while 15.1% were < 5 mm stones in size. The overall mean stone size was 13.2 mm, while the mean sizes of kidneys, ureters, bladder, and urethra were 15.1 mm, 9.3 mm, 18.8 mm, and 13.2 mm, respectively. Single stones were almost 70%, while staghorn calculi were 8.2%. More than 76% of the stones were located in the renal pelvis and middle calyces within the kidney. In the ureter, the stones were almost equally distributed between the proximal, distal, and ureterovesical junction (UVJ). On the other hand, >60% of the patients with urolithiasis showed some degree of hydronephrosis ranging between mild to severe. In total 24% of the patients were asymptomatic. Flank pain and renal colic contained 55%, while abdominal pain and lower urinary tract symptoms (LUTS) were 10% each (see Table 2).

**Table 2:** Overall urolithiasis data demographics, and radiological characteristics.

Characteristics				Frequency (n)	Percent (%)
Gender		Male		702	65.3
		Female		373	34.7
Age (years)		Children (≤17 years)		79	7.3
		Adult (≥18 years)		996	92.7
		Mean and standard deviation (SD)		45.6 (Mean age)	21.1 (SD)
Stone size (mm)		<5		169	15.7
		5–10		356	33.1
		11–20		313	29.1
		21–30		139	12.9
		31–40		55	5.1
		≥41		43	4
		Mean and standard deviation (SD)		13.2 (Mean size)	10.5 (SD)
Affected organ (stone)		Renal		613	57
		Ureter		382	35.5
		Bladder		70	6.5
		Urethra		10	0.9
Laterality	Renal	Right		233	38
		Left		219	35.7
		Bilateral		161	26.3
	Ureter	Right		218	57.1
		Left		132	34.5
		Bilateral		32	8.4
Location	Renal	Upper calyces		31	5.4
		Middle calyces		269	46.6
		Lower calyces		106	18.3
		Renal pelvis		172	29.7
	Ureter	Proximal ureter		106	27.8
		Mid ureter		62	16.3
		Distal ureter		111	29.1
		Ureterovesical junction (UVJ)		102	26.8
No. of stones		Single		745	69.3
		Multiple		330	30.7
Staghorn		Yes		88	8.2
		No		987	91.8
Indication for the CT-scan		Flank pain		335	31.2
		Abdominal pain		113	10.5
		LUTS		106	9.9
		Renal colic		261	24.3
		Asymptomatic		259	24.1
		Hematuria		1	0.1
Hydronephrosis		No		420	39.1
		Yes	Mild	336	31.3
			Moderate	188	17.5
			Severe	131	12.2

The total children (≤17 years) population was 616 (8.5%), of which 79 (12.8%) had urolithiasis with a mean age of 12.3 and 3.5 SD. Male patients were 54.4%, while female patients were 45.6%. Similar to the overall population, renal stones were most predominant accounting for 68.4%. Stone sizes between 11 and 20 mm were 48.1%, while between 5 and 20 mm accounted for 75%. Only 5.1% of the stones in children were below 5 mm in size. Single stones were approximately 59.5%, while multiple stones were 40.5%. Within the pediatric group, there was a 7.6% staghorn calculi in the kidney. In ureter, the majority of the stones (79%) were located in the lower segment of the ureter (distal and UVJ). Moderate and severe hydronephrosis accounted for 34%. Regarding the indication of the scan, flank pain and renal colic were > 63%, while asymptomatic patients were 22.8% (see Table 3).

**Table 3:** Urolithiasis data demographics, and radiological characteristics (<18 years).

Characteristics				Frequency (n)	Percent (%)
Gender		Male		43	54.4
		Female		36	45.6
Age (<18 years)		Mean and standard deviation (SD)		12.3 (Mean age)	3.50 (SD)
Stone size (mm)		<5		4	5.1
		5–10		21	26.6
		11–20		38	48.1
		21–30		13	16.5
		31–40		3	3.8
		≥41		0	0
		Mean and standard deviation (SD)		13.97 (Mean size)	6.89 (SD)
Affected organ (stone)		Renal		54	68.4
		Ureter		19	24.1
		Bladder		4	5.1
		Urethra		2	2.5
Laterality	Renal	Right		26	48
		Left		14	26
		Bilateral		14	26
	Ureter	Right		14	73.7
		Left		5	26.3
		Bilateral		0	0
Location	Renal	Upper calyces		4	8.5
		Middle calyces		17	36.2
		Lower calyces		7	14.9
		Renal pelvis		19	40.4
	Ureter	Proximal ureter		3	15.9
		Mid ureter		1	5.2
		Distal ureter		10	52.6
		Ureterovesical junction (UVJ)		5	26.3
No. of stones		Single		47	59.5
		Multiple		32	40.5
Staghorn		Yes		6	7.6
		No		73	92.4
Indication for the CT-scan		Flank pain		35	44.3
		Abdominal pain		1	1.3
		LUTS		6	7.6
		Renal colic		15	19
		Asymptomatic		22	22.8
		Hematuria		0	0
Hydronephrosis		No		22	27.8
		Yes	Mild	30	38
			Moderate	13	16.5
			Severe	14	17.7

**Figure 1:**
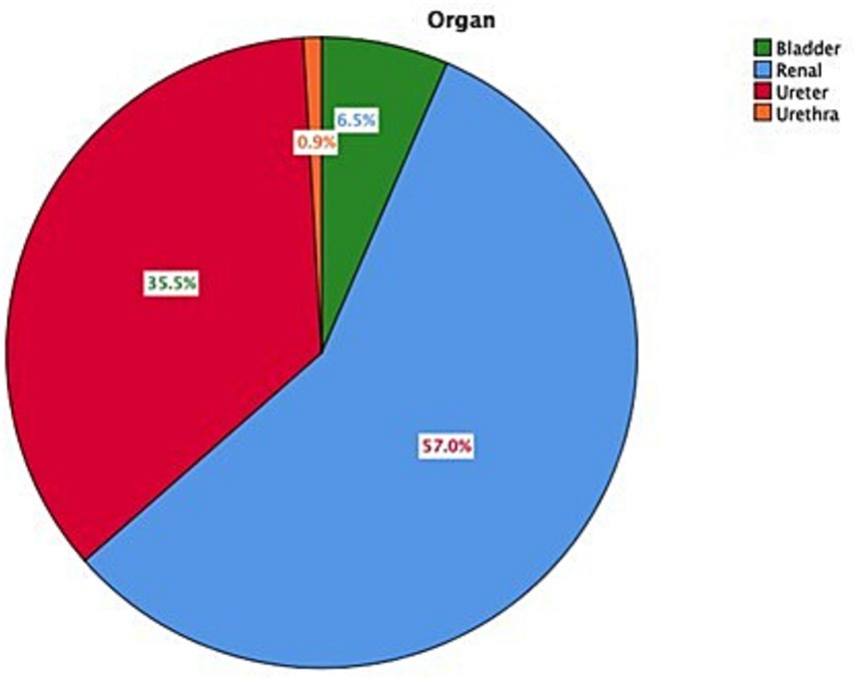
Distribution of the affected organs in patients with urolithiasis.

**Figure 2:**
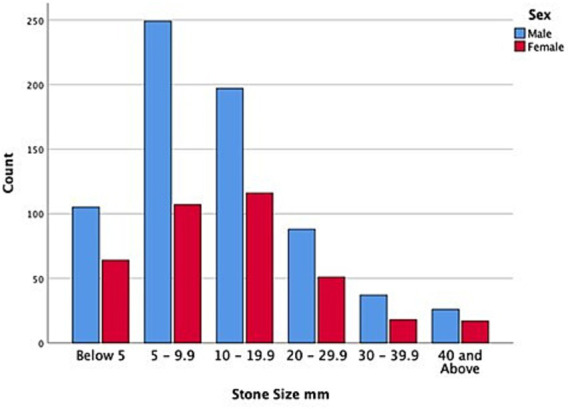
Stone size by gender in patients with urolithiasis.

**Figure 3:**
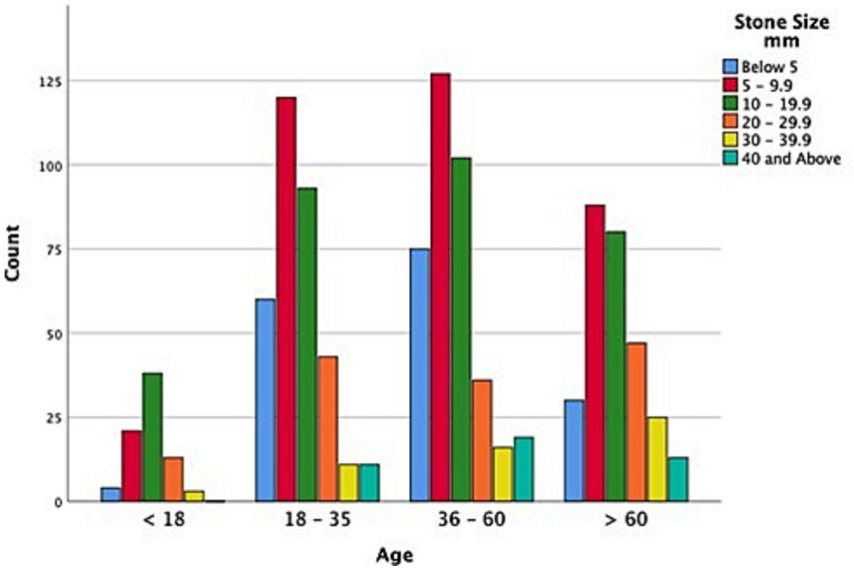
Different age groups and stone sizes of patients with urolithiasis.

**Figure 4:**
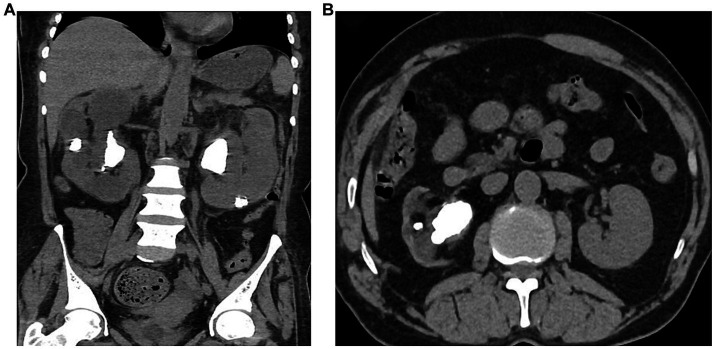
**(A)** Computer reconstructed NCCT of the abdomen showing bilateral multiple renal stones. **(B)** NCCT of the abdomen showing right renal stones.

Figure 1 summarizes the distribution of urolithiasis among different urinary organs, while Figure 2 presents the affected organs by different age groups in which all age categories showed that kidneys were the most predominantly affected. Male patients showed bigger stones in each stone size category when compared with female patients, as shown in Figure 3. Stone sized between 5 and 10 mm was most commonly recorded, followed by the size of 10–20 mm in the four age groups classified (see Figure 4).

## Discussion

Urinary stone disease (urolithiasis) is a major worldwide health burden with high prevalence in many countries (14). Wang et al.’s study delineated the global stone belt with a high prevalence of urolithiasis stretching many regions around the world including Northeastern Africa where Somalia is located (15). In the same study, authors indicated that geogenic factors and climate change has an influence on the stone prevalence, and they presented that some countries have a greater risk of urinary stone disease (15). For instance, Somalia has an estimated 30–35% risk in most parts of the country except northeastern and central regions which showed a higher risk of the disease based on the probability calculations of WofE (weights-of-evidence methodology) (15). Perhaps, our high prevalence of 14.8% could be explained by the abovementioned predictions. Similarly, Somalia though not part of the Afro-Asian stone-forming belt that includes Sudan, it shares similar characteristics with Sudan in terms of lifestyle and diet, especially in the consumption of animal protein as well as the factor of consanguinity that prevails among the majority of the ethnic groups (16). In contrary, urolithiasis etiology and its mechanisms of formation are multi-factorial (17). Calcium oxalate, carbapatite, urate, struvite, and brishite are the most common types in terms of stone composition (17). No study from Somalia is yet to address the stone compositions of the affected populations.

Ultrasound (US) is the basic diagnostic tool that is safe, quick, and cheap with moderate sensitivity and specificity (18). Although the US is considered an essential and effective method for the diagnosis of urinary stones, NCCT is recognized as the gold-standard technique for detecting stones in the urinary system (19). Low-dose NCCT is reported to have 96% sensitivity and 97% specificity with 99% positive and 90% negative predictive values in detecting urinary stones. Consequently, the usage of CT scans has drastically increased over the years; for instance, the annual use of CT abdomen and pelvis in the US has increased from 24.6% in 2006 to 49.4% in 2015 per visit (20). Furthermore, in the emergency department, the upper urinary calculi diagnosis increased from 289 to 306/100,000 people between 2006 and 2009, respectively (21). The use of NCCT has also increased the incidental findings of urolithiasis while examining other abdominopelvic pathologies since small, non-obstructing calyceal stones tend to be silent (22). Although CT scan is believed to be one of the most sensitive methods of kidney stone detection and characterization, other common techniques in use are plane x-ray which occasionally cannot reliably detect non-calcified stones as well as spectroscopy methods which allow for the identification of the chemical nature of stone constituents and their proportions within a calculus (23).

Global urolithiasis prevalence ranges between 1 and 19% in the literature (2, 24). The data regarding urolithiasis prevalence in the region of East Africa and Sub-Saharan Africa are scarce. The current study found a high prevalence of 14.8% of urolithiasis based on the patients who underwent abdominopelvic CT scans. The relatively high prevalence observed in this study could be attributed to the fact that all the major risk factors for urolithiasis include high consumption of animal protein and carbohydrate diet which have all been shown to enhance urinary stone formation abound (25). In addition, there is documented evidence indicating a high prevalence of urinary stone in countries such as Somalia with hot climate (16). This is becoming exacerbated by the prolonged drought periods experienced in the last decade making access to portable drinking water limited thereby increasing the risk for kidney stone formation. However, a recently published retrospective study of 3 years from Mogadishu concerning renal colic containing 435 patients found that 63.4% of the cases had urolithiasis (26). Furthermore, similar authors reported the first pediatric urolithiasis (<18 years) from Somalia, in which they identified 227 children with urolithiasis in the 6-year period (27). The above studies which were done in a single center in such a short period and other case reports as well as studies assessing the clinical characteristics and outcomes of adult patients with urolithiasis all indicate the high incidence of urinary stone disease in the country, which could elucidate the high urolithiasis prevalence in our current study (28, 29).

There are several treatment interventions for the management of urinary stones such as medical expulsive therapy (MET), extracorporeal shock wave lithotripsy (ESWL), retrograde intra-renal surgery (RIRS), and percutaneous nephrolithotomy (PCNL) depending on the stone size and location according to the European Association of Urology (EAU) guideline (28). Fortunately, minimally invasive procedures including ESWL, RIRS, and PCNL have become more popular in recent years in Mogadishu, Somalia, since there are limited centers providing these treatments (26). The estimated annual cost for the treatment of urolithiasis reached 5.3$ billion in 2014 in the US alone compared to 898$ million in 1984 as reported by Ghani et al. (21). However, it is important to note that the economic burden of urinary stones in terms of treatment and diagnosis varies significantly from healthcare system to the other (29). Unfortunately, the financial burden of kidney stone disease is getting exacerbated by the increase in the prevalence of the disease resulting in further economic costs due to direct procedural and medical management costs in addition to the indirect costs to health systems and patients (30). On the other hand, urolithiasis mainly affects labor force of the population between the ages of 18 and 65 years creating huge indirect economic burden on society and the individuals affected by the disease (28). In a study by Saigal et al. showed 30% of the patients will miss the work translating to 19 working hours per year because of their condition while when hospitalized the missing hours more than 50 (28). In the same study, authors estimated $775 million indirect costs per year caused by the stones (28). The current study’s mean age is 45.6 years of age indicating that the disease affects both people’s lives and their productive working hours. Similarly, in the study by Arslan et al., the mean age of renal colic patients from Mogadishu, Somalia, was 36.7 years old (24). A holistic approach to addressing this important health challenge would require the functional healthcare system. Unfortunately, due to the protracted war, the healthcare service has been severely damaged. In addition, another important factor that tends to increase the risk of urolithiasis in Somalia is the frequent droughts that make access to water very difficult, especially among the rural and displaced population. Notwithstanding, members of the population can adjust their dietary habits and other nutritional requirements while hoping that the government continues its efforts to build a functional healthcare delivery system.

The kidneys are the predominantly affected organs according to worldwide existing literature (6); similarly, our current data revealed that 57% nephrolithiasis was followed by ureterolithiasis which accounted for 35%. The vast majority of the global statistics on urolithiasis show that male patients are frequently affected than female patients (8). Correspondingly, this study demonstrated 18.7% male-specific urolithiasis prevalence while female patients were 10.6%. On the other hand, patients with hydronephrosis in the present study accounted for more than 60%, and this back pressure to the kidney independently has a tendency to increase the risk of renal damage and acute kidney injury (AKI) (29). Furthermore, there are several factors to consider when planning stone management such as stone size, conventionally the higher the stone size, the more complications it involves (27, 31, 32). In the present study, we found that 29.1% of the stones were between 10 and 20 mm, while 21% were > 20 mm, necessitating surgical interventions and could potentially increase the risk of complications. The presence of multiple stones and staghorn-type calculi are other factors increasing the severity of the disease (33, 34); in our study, multiple stones and staghorn calculi were 30.7 and 8.2%, respectively.

Children are special population that the disease affects, and their prevalence ranges between 4 and 10% (35). Similarly, in the present study, 79 (12.8%) children had urinary stones with which nephrolithiasis accounted for 68.4%. With respect to bladder stones specifically, while 6.2% (63) of the adults had bladder stones, only 0.4% (4) of the children had bladder stones. Global data also show that nephrolithiasis is most predominant in children (36). Approximately 70% of the children in the study had >10 mm stones in their urinary system. The latter necessitates investing more in surgical intervention methods in children for early treatment and ultimately better outcomes.

One of the major limitations of this study is the fact that it is a retrospective hospital-based study and hence may lack generalizability in the overall population, especially as many other factors including environment, lifestyle, and economic status could all play a role in the condition. Furthermore, the limited clinical and demographic data in the records reviewed are another limitation in our study we need to acknowledge. Notwithstanding, the information provided will contribute to understanding of the conditions among residents in Mogadishu.

In conclusion, a CT scan-based urolithiasis prevalence is 14.8% in Mogadishu, Somalia, and these results are consistent with the probability calculation of the WofE (weights-of-evidence methodology) based on several risk factors including temperature, climate change, mineral deposit, drinking water quality, and distribution of carbonated rocks. Considering the high prevalence of the disease, Somalia should invest more in prevention and treatment facilities while also training urologists who are capable of utilizing minimally invasive techniques in the country.

## Data availability statement

The raw data supporting the conclusions of this article will be made available by the authors, without undue reservation.

## Ethics statement

The studies involving humans were approved by SIMAD University Ethical Review Board. The studies were conducted in accordance with the local legislation and institutional requirements. Written informed consent for participation in this study was provided by the participants’ legal guardians/next of kin.

## Author contributions

ND: concept, manuscript writing, and editing. MA: data analysis. BG and HD: manuscript editing. MS: concept and design. FM and AM: data collection and organizing. JH: manuscript English correction and editing. All authors contributed to the article and approved the submitted version.
